# Rates and Patterns of First-Time Admissions for Acute Coronary Syndromes across Western Australia Using Linked Administrative Health Data 2007–2015

**DOI:** 10.3390/jcm10010049

**Published:** 2020-12-25

**Authors:** René Forsyth, Zhonghua Sun, Christopher Reid, Rachael Moorin

**Affiliations:** 1 Discipline of Medical Radiation Sciences, Curtin University, Perth, WA 6102, Australia; rene.forsyth@postgrad.curtin.edu.au; 2 School of Public Health, NHMRC Centre of Research Excellence in Cardiovascular Outcomes Improvement, Perth, WA 6102, Australia; christopher.reid@curtin.edu.au; 3 Centre of Research Excellence in Therapeutics, Monash University, Melbourne, VIC 3800, Australia; 4 School of Public Health, Curtin University, Perth, WA 6102, Australia; R.Moorin@curtin.edu.au; 5 School of Population and Global Health, the University of Western Australia, Crawley, WA 6009, Australia

**Keywords:** acute coronary syndrome, Western Australia, rates of admission, inter-hospital transfers, linked data, percutaneous coronary intervention

## Abstract

Acute coronary syndrome (ACS) is globally recognised as a significant health burden, for which the reduction in total ischemic times by way of the most suitable reperfusion strategy has been the focus of national and international initiatives. In a setting such as Western Australia, characterised by 79% of the population dwelling in the greater capital region, transfers to hospitals capable of percutaneous coronary intervention (PCI) is often a necessary but time-consuming reality for outer-metropolitan and rural patients. Methods: Hospital separations, emergency department admissions and death registration data between 1 January 2007 and 31 December 2015 were linked by the Western Australian Data Linkage Unit, identifying patients with a confirmed first-time diagnosis of ACS, who were either a direct admission or experienced an inter-hospital transfer. Results: Although the presentation rates of ACS remained stable over the nine years evaluated, the rates of first-time admissions for ACS were more than double in the rural residential cohort, including higher rates of ST-segment elevation myocardial infarction, the most time-critical manifestation of ACS. Consequently, rural patients were more likely to undergo an inter-hospital transfer. However, 42% of metropolitan admissions for a first-time ACS also experienced a transfer. Conclusion: While the time burden of inter-hospital transfers for rural patients is a reality in health care systems where it is not feasible to have advanced facilities and workforce skills outside of large population centres, there is a concerning trend of inter-hospital transfers within the metropolitan region highlighting the need for further initiatives to streamline pre-hospital triage to ensure patients with symptoms indicative of ACS present to PCI-equipped hospitals.

## 1. Introduction

Cardiovascular disease (CVD) represents a significant global burden, annually killing more people than any other cause [1]. In Australia alone, it was estimated that 59,100 people aged 25 years and over experienced an acute coronary syndrome (ACS) event in 2017 [2], with Australia’s population for the same year estimated to be 24.6 million [3]; equating to approximately 2.4 ACS events per 1000 person–years. Worldwide data collection on ACS utilises a variety of methods. Cardiac registries such as the CONCORDANCE Registry in Australia [4]; the ACS Snapshot Study of Australia and New Zealand [5]; the All New Zealand Acute Coronary Syndrome-Quality Improvement (ANZACS-QI) [6] and the National Cardiovascular Data Registry (NCDR) of the American College of Cardiology [7] enrol representative hospitals or clinics to inform on clinical characteristics, management and outcomes for ACS hospitalisations. Similarly, government agencies gather data on the numbers of ACS hospitalisations for a given time period using admission statistics. Both methods offer advantages and disadvantages. Cardiac registries provide in depth data on clinical characteristics and outcomes that cannot be derived from hospital admission data alone. However, these are often based on the voluntary participation of hospitals or clinics in the registries and often are for a short snapshot of time rather than an ongoing, whole population cohort [5,8]. Conversely, hospital admission data can capture more accurate rates of ACS hospitalisations, but can lack in depth clinical data necessary to inform policy and can be prone to double-counting, secondary to inter-hospital transfers [9].

The Western Australian Data Linkage System (WADLS) [10] provides anonymised population-based records using hospital admission data (for both public and private hospitals), public emergency department data and mortality records all linked via probabilistic matching allowing patients to be followed throughout multiple hospitalisations for related conditions based on the International Classification of Diseases (Australian Modification) coding.

The diagnosis of the type of ACS requires interpretation of an electrocardiogram (ECG) for the critical distinction between the ST-segment elevation myocardial infarction (STEMI) and non-ST-segment elevation acute coronary syndromes (NSTEACS) which is further categorised into non-ST-segment elevation myocardial infarction (NSTEMI) or unstable angina (UA), based on patient history, examination and cardiac enzyme biomarkers [11]. Early diagnosis of STEMI as well as the appropriate assessment and risk stratification of NSTEACS, are essential to facilitate early reperfusion by primary percutaneous coronary intervention (PCI), coronary artery bypass grafting (CABG), pharmacotherapy or fibrinolytic therapy for improved morbidity and mortality [11,12,13,14]. 

The challenge for some parts of the world such as Western Australia (WA), is the remoteness of a large section of the community outside capital cities. The estimated resident population of WA was 2.58 million people in 2017 of which 92% live in the South West Land Division and more specifically 79% living in the greater capital city region, leaving the rest of the state sparsely populated [15]. In addition, current service provisions for ACS are predominantly centred in the capital city area with three tertiary hospitals, several secondary and private hospitals with round-the-clock catheterisation laboratories and one regional centre in the South West offering coronary intervention for planned admissions [16]. It is not feasible to expect advanced cardiac catheterisation laboratory (CCL) facilities in every hospital across the world, necessitating inter-hospital transfers to facilities capable of PCI, where indicated. Furthermore, in the rural setting, the lack of advanced medical facilities is further compounded by staff shortages and variable workforce experience and skills [16]. However, these centres have provisions for the administration of fibrinolytic therapy prior to emergent or elective transfer for PCI, known as facilitated or rescue PCI. In more metropolitan settings, strategies to ensure ambulance ECG with transmission to a cardiac facility for the evaluation of STEMI is essential to ensure that ambulances can bypass secondary hospitals in favour of PCI-capable facilities wherever possible in the metropolitan region and prevent the inherent delays associated with triage and inter-hospital transfer (IHT) [17,18]. 

The aim of this study was to evaluate the whole-of-population rates of admission for first-time presentations of ACS in Western Australia (WA), accounting for the number of inter-hospital transfers, over a period of nine years using Linked Administrative Data from the WA Data Linkage System (WADLS) [10] and Population Census Data from the Australian Bureau of Statistics (ABS) [19].

## 2. Methods

This paper follows the Reporting of Studies Conducted Using Observational Routinely-Collected Health Data (RECORD) Statement [20].

### 2.1. Data Sources

Data from the WA Hospital Morbidity Data System (HMDS), WA Emergency Department Data Collection (EDDC) and WA Death Registrations from 1 December 2007 to 31 December 2015, with further data to establish a look-back period from the HMDS available from 01 January 2002 were linked by the WADLS. The WADLS uses routinely collected, longitudinal, whole-population, administrative health and medical data with linkages obtained through key demographic information including name, date of birth, home address and hospital medical record numbers. Privacy is ensured by linking records from different datasets using pre-established linkage keys, removing identifiable demographic data and providing only non-identifiable demographic data such as month and year of birth, sex and postcode; in addition content data describing what happened to the person during each record, such as diagnosis and treatment codes [10]. The WADLS provides data under a waiver of consent due to the de-identified nature of the data and the research team is required to work under strict conditions to ensure the security of the data.

Routinely collected data include socio-economic data (date of birth; sex; postcode of residence; Socio-Economic Indexes for Areas (SEIFA) [21]) and clinical service utilisation data for each hospital admission, namely the admission date and time; separation (discharge) date and time; primary, secondary and co-diagnoses (up to 20 fields); primary and up to ten secondary interventions including procedural dates; hospital region; admission status (emergency presentation or elective) and insurance status (private or public).

Population data were sourced from the Australian Bureau of Statistics (ABS) [19] and reported age groups were determined so as to align with the available ABS age groups. All-cause hospital separations were sourced from the Australian Institute of Health and Welfare [22,23] to evaluate the rate of hospitals for ACS compared to all separations for the same time period.

### 2.2. Population and Ascertainment of ACS Events

The cohort consisted of all individuals admitted into a WA hospital having a first-time record indicating a primary diagnosis of ACS between 1 January 2007 and 31 December 2015, identified using the International Classification of Diseases, Australian Modification (ICD-10-AM) codes [24] (Appendix A). First-time ACS admissions were defined as individuals with no records indicating a primary or secondary diagnosis of ACS prior to 1 January 2007, using HMDS and EDDC data from 1 January 2002 as a look-back period. Individuals who had ACS events prior to the commencement of the study period (i.e., 1 January 2007) were excluded from the cohort. Subsequent admissions for patients after their incident event were included as readmission events if they were in relation to a primary or secondary diagnosis of ACS.

### 2.3. Patient Characteristics

Sex, identification of being of Aboriginal or Torres Strait decent and date of birth were coded directly within the data. Reported ages were then categorised based on the age of the patient at the time of their incident ACS admission to match available population data. Residential postcodes were used to determine the health region of residence and SEIFA. The primary and secondary diagnosis fields were used to identify those patients admitted for ACS with the remaining 20 co-diagnosis fields used to identify specific comorbid conditions during the same episode of care. These data were also used to ascertain total comorbidity using the Multipurpose Australian Comorbidity Scoring System (MACSS) [25] at one year and five years prior to the incident event. A summary of the diagnosis codes used to define comorbidity is shown in Appendix B. In the event a patient was diagnosed with two or more types of ACS within the same episode of care, the method of diagnosis hierarchy previously validated by Sanfilippo et al. [26] and Lopez et al. [27] was applied where the final diagnosis was the most severe diagnosis; with STEMI being the most severe, followed by NSTEMI and lastly UA. The determination of the interventions performed was based on The Australian Classification of Health Interventions, Tenth Revision from the National Centre for Classification in Health [28] (Appendix A).

The EDDC was combined with the HDMS to identify those patients who presented to an emergency department and were admitted at the same hospital, versus those patients who were transferred to another hospital. Distinction between IHT during the same episode of care, as opposed to a readmission, were identified by a secondary HMDS record within 24 h of the time of separation, where the method of patient discharge and discharge destination from the first hospital indicated a transfer. A new admission beyond 24 h post discharge and in the absence of the aforementioned discharge indicators within 24 h, the record was assigned as a readmission. 

Emergency versus elective admissions are coded directly in the HMDS. In-hospital mortality, including the date of death, is coded within the HMDS; while post-discharge mortality, including data of death and cause of death (COD) based on ICD-10-AM coding, was determined using the linked mortality dataset. Reported time frames calculated between the date of separation for the first admission and the date of death. The ICD-10-AM was used to categorise the COD into acute myocardial infarction (AMI), other ischemic heart diseases (IHD) excluding AMI and non-IHD causes for 0 to 30-day, 30-day to 1-year and beyond 1-year mortality.

### 2.4. Statistical Analysis

Unadjusted estimates of the person’s time at risk were calculated using the number of persons residing in WA in each age bracket (18–39, 40–49, 50–59, 60–69, 70–79 and 80+ years) on 30th June for each year from the ABS as the denominator. The Australian Institute of Health and Welfare data on annual separation rates were used as the denominator to calculate the rate of first-time ACS separations versus all-cause hospital separations. Categorical data were reported as frequencies and percentages and continuous data were reported as the means, standard deviations and ranges. Where appropriate, categorical data were also presented as rates, using the admitted cohort as the denominator unless otherwise specified. Confidence intervals were calculated using a confidence interval calculator for single incidence rates with the two-sided confidence level assigned at 95% [29]. The Mann–Whitney *U* test and the Pearson chi-squared tests were used for between group analyses. Statistical significance was assigned at the level of *p* < 0.05. All analyses were performed using IBM SPSS Statistics for Windows, Version 25.0 [30] and Stata Statistical Software: Release 16 [31].

## 3. Results

**Figure 1:**
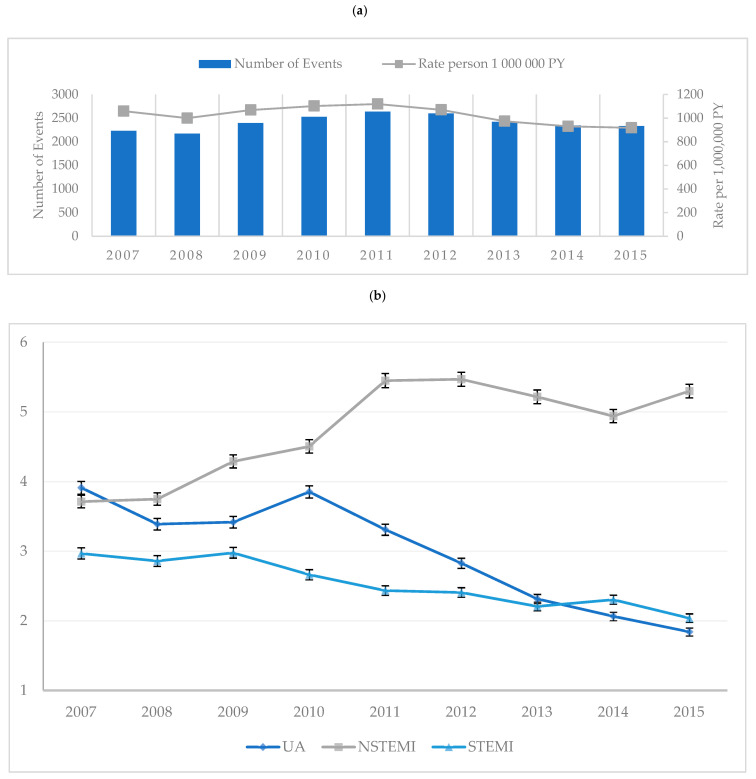
Rates of all first-time admissions for acute coronary syndrome in Western Australia between 1 January 2007 and 31 December 2015. (**a**): Number and rates of first-time admission for acute coronary syndrome per 1,000,000 person–years. (**b**): Rates of first-time admissions by acute coronary syndromes per 10,000 person–years (PY) with confidence intervals. UA: unstable angina; NSTEMI: non-ST-segment elevation myocardial infarction; STEMI: ST-segment elevation myocardial infarction.

**Figure 2:**
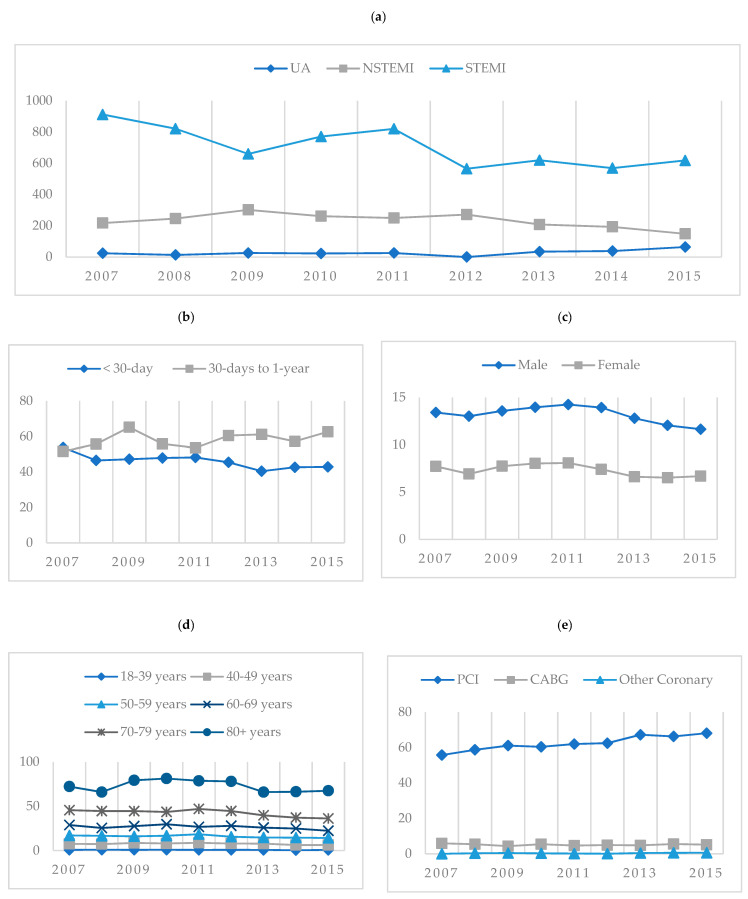
Rates of all first-time admissions for acute coronary syndrome in Western Australia between 1 January 2007 and 31 December 2015. (**a**): In-hospital mortality rates of first-time admissions by acute coronary syndromes per 10,000 person–years. (**b**): Mortality rates for first-time admissions by acute coronary syndrome per 1000 person–years. (**c**): Rates of first-time admissions per 10,000 person–years by sex. (**d**): Rates of first-time admissions per 10,000 person–years by age. (**e**): Rates of PCI including one or more stent insertions; CABG and other coronary procedures. UA: unstable angina; NSTEMI: non-ST-segment elevation myocardial infarction; STEMI: ST-segment elevation myocardial infarction; PCI: percutaneous coronary intervention including one or more stent insertions; CABG: coronary artery bypass grafting; Other Coronary: other procedures of the coronary artery not otherwise specified; MACSS: Multipurpose Australian Comorbidity Scoring System.

Rates of admissions for the first-time diagnoses of ACS remained steady throughout the nine-year study timeframe averaging ten per 10,000 person years with an average of 2405 admissions per calendar year (Figure 1a) and 21,648 total admissions between 1 December 2007 and 31 December 2015 (Appendix C). However, rates of UA and STEMI decreased from 2010 to 2015 as NSTEMI rates showed an increase. Annual rates of UA showed a statistically significant difference from 2010, decreasing with each subsequent year. Rates of STEMI presentations were statistically significantly lower between 2009 and 2011 and between 2014 and 2015. Rates of NSTEMI steadily increased with each year from 2010 and although not always a statistically significant change per year within NSTEMI, this condition does contribute statistically significantly higher rates of the ACS burden from 2009 compared to UA and STEMI. (Figure 1b). In-hospital mortality rates were stable for UA and NSTEMI with a slow decline noted for STEMI patients (Figure 2a) and the overall all-cause mortality rate at 30 days also remained stable at an average of 459 per 10,000 person–years (PY), increasing to an average of 583 per PY for 30-day to 1-year mortality (Figure 2b). The rates of first-time admissions were higher in males and increased with each 10-year incremental rise in age (Figure 2c,d). Rates of PCI including one or more stent insertion increased from 56 procedures per 100 PY in 2007 to 68 per 100 PY for every confirmed diagnosis of ACS, with rates of CABG contributing approximately five procedures per 100 PY per annum over the nine years (Figure 2e). The data for Figure 1 are tabulated in more depth in Appendix A.

**Figure 3:**
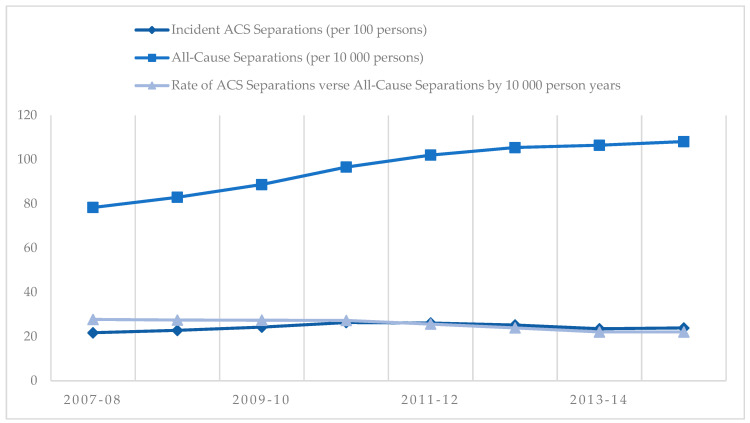
Rates of incident acute coronary syndrome (ACS) separations versus all-cause separations in Western Australian between 1 January 2007 and 31 December 2015 financial years.

**Figure 4:**
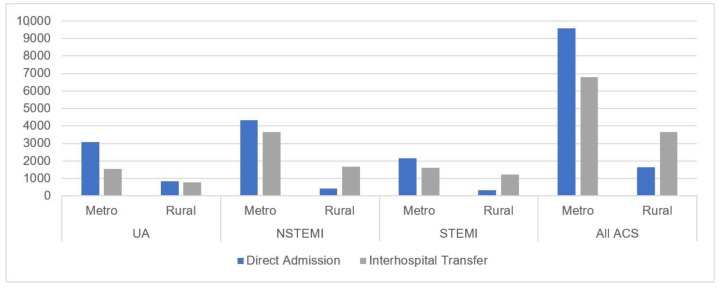
Number of direct admissions versus inter-hospital transfers for acute coronary syndromes in Western Australia between 1 January 2007 and 31 December 2015 by metropolitan and rural residence. UA: unstable angina; NSTEMI: non-ST-segment elevation myocardial infarction; STEMI: ST-segment elevation myocardial infarction.

Rates of incident (first-time) ACS separations remained stable as all-cause hospital separations increased, resulting in an average rate of 25 admissions per 10,000 PY, or 0.25% of all admission attributable to first-time ACS events annually (Figure 3). A higher number of metropolitan patients were admitted directly for care compared to rural patients in the NSTEMI and STEMI cohorts with similar numbers of direct admission and inter-hospital transfer in the UA cohort (Figure 4). 

As demonstrated in Table 1, two-thirds of ACS admissions were male with a mean age of 65 years and 63 years for metropolitan and rural patients, respectively. The mean age for rural female patients was 66 years which is statically significantly younger than metropolitan female admissions with a mean age of 72 years (*p* < 0.001). More rural admissions were identified as being of Aboriginal and/or Torres Strait Islander descent at 14% compared to 2% of metropolitan admissions and two-thirds of rural patients were from the high- and highest-disadvantaged SEIFA compared to nearly 50% of metropolitan admissions, which pertained to individuals living in the least or less disadvantaged areas. Metropolitan patients used emergency services more frequently than rural patients (50% and 35%, respectively). NSTEMI accounted for the highest proportion of primary diagnoses with STEMIs at 23% and 29% for metropolitan and rural admissions, respectively. More than half of all metropolitan admissions presented directly to the treating hospital (58%) whereas 69% of rural patients experienced a transfer. 

Table 2 presents the rates of primary diagnosis, principal procedure, inter-hospital transfers and in-hospital and post-discharge mortality between the metropolitan and rural cohorts. The rate of admissions for STEMI was higher in rural patients at 29.3 admissions per 100 PY of all ACS admissions, while NSTEMI presentations were higher in the metropolitan cohort. Metropolitan admissions also had a higher rate of PCI and lower rates of inter-hospital transfer. All-cause in-hospital mortality was statistically significantly higher in the metropolitan cohort at 3.1 deaths per 100 PY. The highest rate of 30-day mortality was due to AMI causes at 46 deaths per 100 PY, which was particularly high in the rural cohort at 51 deaths per 100 PY compared to 45 deaths in the metropolitan group, although these figures showed no statistically significant difference.

A higher proportion of transferred patients was diagnosed with NSTEMI and only 1% more STEMI cases compared to direct presentations, as shown in Table 3. The rate of patients to have a PCI including one or more stent insertion was 57 per 100 PY for non-transferred patients and 72 per 100 PY for admissions that incorporated an inter-hospital transfer. Transferred patients had slightly lower rates of comorbidities diagnosed during the same episode of care, with all but obesity statistically significantly different between the two groups. The rate of in hospital mortality was significantly higher in non-transferred patients as was the rate of post-discharge mortality at both 30 days and 30 days to 1 year. However, the rate of mortality occurring more than 1 year post discharge was higher in the transferred group. Post-discharge mortality rates for non-ischemic heart disease and AMI were higher in transferred patients.

## 4. Discussion

The construct of the right patient–right treatment–right time is not new in ACS reporting. Rates of first-time admission for ACS were more than double in the rural residential cohort compared to metropolitan patients with disparities in health conditions and outcomes for rural patients recognised globally and within Australia [9,32,33]. One of the largest challenges facing a rural patient in the event of ACS is access to care in a timely manner from symptom onset, with clear guidelines for the treatment of STEMI, depending on access to a PCI-capable hospital, inter-hospital transfer travel times, and a patient’s suitability for PCI, CABG or fibrinolytic therapy [34]. In our study, nearly 80% of rural admissions for STEMI and NSTEMI were transferred, which is not a surprising finding given the unavailability of PCI-capable hospitals in rural WA. However, of greater concern is the proportion of metropolitan patients also being transferred during the episode of care. With 54% and 24% of NSTEMI and STEMI patients being transferred, respectively, of which 80% go on to have PCI including one or more stent insertion or CABG. The era of ambulance ECG, rapid assessment of chest pain or less common symptoms of AMI en route to the hospital means that STEMI patients can be triaged and bypass non-PCI-capable hospitals. This should result in fewer inter-hospital transfers, particularly for in metropolitan STEMI patients, consequently reducing time to definitive treatment [34,35]. Previous research [18,34,36] has also indicated that inter-hospital transfers in a metropolitan cohort is an ongoing reality and suggests that more focus needs to be put into a coordinated pre-hospital framework to ensure STEMI patients are rapidly identified, allowing for ambulance diversion to an appropriate PCI-capable facility [37]. In comparison, NSTEMI diagnoses, often made more complicated by the requirement of serial troponins in addition to ECG and detailed clinical history, require risk stratification to determine the need for invasive or conservative treatment at an appropriate time interval from symptom onset [11,38]. Consequently, inter-hospital transfers in the presentation of NSTEMI may remain a necessity after risk stratification with previous literature indicting little long-term benefits of early invasive strategies for NSTEACS patients without recurrent symptoms and a low risk of further ischemic events [11,38,39]. 

Although the overall rate of ACS presentations in our study remained stable over the nine-year time frame, rates of NSTEMI increased with each year with decreasing rates of UA while STEMI presentations remained steady. This trend has previously been reported in the Department of Health’s Model of Care for Acute Coronary Syndromes in WA [16] which attributed an apparent decline in angina presentations with a correlating increase in NSTEMI presentations between 1999 and 2008 based on sensitive and specific biomarkers of myocardial injury, primarily troponins, resulted in a decline in angina diagnoses in favour of NSTEMI. The same trend is noted in our study from 2010 onwards, which could be partially explained by the uptake time of the new definitions in the WA health system. 

We found that in-hospital all-cause mortality was higher for direct presentations compared with transferred patients. Although counterintuitive given what is known about longer infarct times due to the postponement of reperfusion to facilitate an inter-hospital transfer to a PCI-capable hospital, this finding concurs with previously published randomised controlled trials citing similar results [34,40,41,42]. Kawecki et al. [34] theorised that this may be due to the longer delay associated with an inter-hospital transfer resulting in more patients dying prior to arrival at a PCI-capable hospital compared to their direct counterparts, thereby positively pre-selecting the cohort eligible for analysis (a form of immortal time bias) [42]. The same pattern can be noted for death within 30 days post discharge, however, the rate of death between 30 day and 1 year are almost identical between transferred and non-transferred patients. After the first year post discharge, transferred patients experience a higher mortality rate at 5 more deaths per 100 PY compared to the non-transferred cohort, indicating a poorer outcome. While differences in comorbidity between transferred and non-transferred patients could explain this, we found that patients with important comorbid conditions such as diabetes, heart failure, congestive pulmonary disease, renal insufficiency, obesity and cardiac arrest were less likely to undergo an inter-hospital transfer. Thus, in our study, confounding due to comorbidity is likely to lead to worse outcomes for direct admissions, hence our estimation of the difference in post 1-year death rates resulting from inter-hospital transfer is likely conservative. Due to the anonymisation of the data, it is unknown if these direct admissions were at a PCI-capable hospital as the first port of call. However, given what is known about the comorbid conditions and rates of invasive treatments, there are two likely explanations for the differences of observed comorbidity between direct and transferred patients. The first is the patient is too unwell to undergo invasive angiography and therefore there is no need for a transfer to a PCI-capable facility. The alternative option is due to the nature of the patient’s comorbidities they may likely be deemed at higher risk and are therefore taken directly to a tertiary facility with PCI-capable faculties, as a matter of triage. 

A key strength of this study is the identification of first-time events. This study assigned incident events of ACS if the patient was admitted for ACS during the 9-year period if the same individual had no prior admission for ACS from 1 January 2002. This ensured prevalent individuals could be removed from the analysis and allowed for accurate follow-up time periods for post-discharge mortality. 

A further strength of linked administrative data is the ability to overcome the challenges associated with over-counting due to inter-hospital transfers which can plague other cardiac registries, which gives WA a unique position to track and monitor trends in ACS admissions.

Although this study has the strength of a large sample size using whole-of-population data during the study time frame, it is not without limitations. The analysis of linked administrative health data is an important tool for the longitudinal tracking of patients over time due to a lack of loss to follow up a complete capture of the population using health services. However, long-term (i.e., greater than one year) outcomes can only be evaluated if sufficient data are available beyond each year or composite end point. Furthermore, the WADLS has no current capacity to track patients who are admitted for the same condition in another Australian State or Territory. However, the data can lack some clinical detail which would create a more holistic approach which cardiac registries could overcome. This includes, but is not limited to, the time of symptom onset, time of first medical contact, whether an ECG was performed en route to the hospital, records of serial troponin levels over time, details of any administered pharmaceuticals, including fibrinolytic medications, accurate arrival times and times of balloon inflation in the cardiac catheterisation laboratory. The addition of these variables to a study such as this would allow for the adherence to the evidence-based guidelines developed by the NHFA/CSANZ to be assessed [11]. While the overall rates of admission for ACS can be reported via linked data, smaller internal registries can inform where the overall delays patients experienced, be it direct presentations or inter-hospital transfers. To overcome the limitations of the data, an aligned previous study by Forsyth et al. [18] used data from a single Perth metropolitan tertiary (teaching) hospital with round-the-clock PCI facilities which recorded key time points as part of their internal auditing process. This study highlighted the significantly longer infarct times for patients who required an inter-hospital transfer for treatment, despite the pre-hospital activation of the CCL at the treating hospital and shorter door-to-balloon times at the treating hospital, compared to direct presentations. Given 43% of Perth metropolitan admissions for first-time presentations of STEMI require an inter-hospital transfer, this is a large volume of patients experiencing a longer time-to-treatment burden and having poorer long-term outcomes.

Finally, this descriptive study is limited as the association between transfer status and patient outcomes was not evaluated. Rather, it sought to determine, at the population level, the rate of all first-time admissions for ACS in WA including the numbers of inter-hospital transfers according to geographic location and patient characteristics. Therefore, results reporting mortality rates should be regarded as exploratory in nature and will be the subject to further evaluation using appropriate modelling and/or quasi-experimental methods. 

## 5. Conclusions

This paper provides information about the rates of ACS presentations in WA hospitals between 2007 and 2015, according to health, region, age and type of ACS. Accurate presentation numbers are essential to monitor the burden of ACS across the world informing researchers and governments alike on disparities between health regions and service utilisation. Of particular concern in our study is the percentage of metropolitan patients still requiring inter-hospital transfers for PCI, despite efforts to ensure STEMI presentations present directly to PCI-capable hospitals. With 48% of all patient admissions during the study period undergoing an inter-hospital transfer during their first admission for ACS, previously reported ACS presentation figures should be interpreted with care as they may be reporting total admission figures, not taking into account inter-hospital transfers during the same episode of care.

## Figures and Tables

**Table 1:** Characteristics and outcomes of first-time admissions for acute coronary syndrome among metropolitan and rural Western Australians, based on residential postcode at the time of admission between 1 January 2007 and 31 December 2015.

Variables	Metro	Rural	Total	Sig *
	*n* (% *)	*n* (% *)	*n* (% *)	*p* < 0.05
	16,357 (75.6)	5290 (24.4)	21,647	
Demographics				
Male Age, Mean (std dev) (range)	65.05 (13.698) (18–101)	62.54 (14.065) (18–99)	64.43 (13.832) (18–101)	0.056
Female Age, Mean (std dev) (range)	72.37 (14.149) (18–103)	65.58 (15.137) (22–101)	70.77 (14.673) (18–103)	<0.001
Male	10,519 (64.3)	3484 (65.9)	14,003 (64.7)	0.040
Indigenous Australian	310 (1.9)	713 (13.5)	1023 (4.7)	<0.001
Socio-Economic Indexes for Areas (SEIFA)				
Highest Disadvantage	1737 (10.6)	1476 (27.9)	3213 (14.8)	<0.001
High Disadvantage	3951 (24.2)	2169 (41.0)	6120 (28.3)	
Moderate Disadvantage	2841 (17.4)	940 (17.8)	3781 (17.5)	
Less Disadvantage	2679 (16.4)	549 (10.4)	3228 (14.9)	
Least Disadvantage	5149 (31.5)	156 (2.9)	5305 (24.5)	
Comorbidities during the Same Admission				
Diabetes	3043 (18.6)	1064 (20.1)	4107 (9.0)	0.015
Cardiac Arrest	238 (1.5)	65 (1.2)	303 (1.4)	0.223
Heart Failure	1832 (11.2)	445 (8.4)	2277 (10.5)	<0.001
Chronic Pulmonary Disease	112 (0.7)	37 (0.7)	149 (0.7)	0.910
Renal Insufficiency < 29 mL/min	412 (2.5)	127 (2.4)	539 (2.5)	0.632
Obese	344 (2.1)	132 (2.5)	476 (2.2)	0.091
Mode of Arrival to 1st Hospital or ED				
Ambulance/Royal Flying Doctor Service	8133 (49.7)	1848 (34.9)	9981 (46.1)	<0.001
Private/Public Transport	7575 (46.3)	3242 (61.3)	10,817 (50.0)	
Other	448 (2.7)	79 (1.5)	527 (2.4)	
Unknown	201 (1.2)	121 (2.3)	322 (1.5)	
Region of 1st Hospital of Admission				
North Metro	6012 (36.8)	496 (9.4)	6508 (30.1)	<0.001
East Metro	5248 (32.1)	738 (14.0)	5986 (27.7)	
South Metro	4880 (29.8)	270 (5.1)	5150 (23.8)	
South West	48 (0.3)	1321 (25.0)	1369 (6.3)	
Great Southern	16 (0.1)	540 (10.2)	556 (2.6)	
Wheatbelt	7 (0.0)	239 (4.5)	246 (1.1)	
Goldfields	17 (0.1)	485 (9.2)	502 (2.3)	
Midwest	27 (0.2)	532 (10.1)	559 (2.6)	
Pilbara	78 (0.5)	328 (6.2)	406 (1.9)	
Kimberley	24 (0.1)	341 (6.4)	365 (1.7)	
Transfer Status by Primary Diagnosis				
Transfer for UA	1532 (33.3)	780 (47.7)	2312 (37.1)	<0.001
Transfer for NSTEMI	3645 (45.7)	1670 (79.3)	5315 (52.7)	
Transfer for STEMI	1610 (42.6)	1212 (78.1)	2822 (53.0)	

% *: the total percent for each cohort (e.g., Metro); Sig *: statistical significance.

**Table 2:** Rates of primary diagnosis, principal procedure, inter-hospital transfers and mortality per 100 person–years for first-time admissions for acute coronary syndrome for metropolitan and rural Western Australians, based on residential postcode at the time of admission between 1 January 2007 and 31 December 2015.

Variables	Metro	Rural	Total	Sig *
	*n* (R *)	*n* (R *)	*n* (R *)	*p* < 0.05
Primary Diagnosis (Total Events)	(*n* = 16,357)	(*n* = 5290)	(*n* = 21,647)	
UA	4603 (28.1)	1634 (30.9)	6237 (28.8)	<0.001
NSTEMI	7978 (48.8)	2105 (39.8)	10,083 (46.6)	
STEMI	3776 (23.1)	1551 (29.3)	5327 (24.6)	
Percutaneous Coronary Interventions (PCI) (Total Events)	(*n* = 16,357)	(*n* = 5290)	(*n* = 21,647)	
Other Coronary Procedures	48 (0.3)	11 (0.2)	59 (0.3)	<0.001
PCI +/− Stent	10,394 (63.5)	3150 (59.5)	13,544 (62.6)	
Coronary Artery Bypass Grafting	819 (5.0)	292 (5.5)	1111 (5.1)	
Inter-Hospital Transfer Status (Total Events)	(*n* = 16,357)	(*n* = 5290)	(*n* = 21,647)	
Direct/No IHT	9570 (58.5)	1628 (30.8)	11,198 (51.7)	<0.001
Yes, IHT	6787 (41.5)	3662 (69.2)	10,449 (48.3)	
In-Hospital Mortality—All Cause (Total Events)	(*n* = 16,357)	(*n* = 5290)	(*n =* 21,647)	
Yes, Died in Hospital	509 (3.1)	117 (0.7)	626 (3.8)	0.001
Mortality—Non-IHD COD (Total Events)	(*n* = 2848)	(*n* = 806)	(*n* = 3654)	
30-Day Mortality	326 (11.4)	97 (12.0)	423 (11.6)	0.804
30-Day to 1-Year Mortality	650 (22.8)	189 (23.4)	839 (23.0)	
More than 1-Year Mortality	1872 (65.7)	520 (64.5)	2392 (65.5)	
Mortality—AMI COD (Total Events)	(*n* = 854)	(*n* = 240)	(*n* = 1094)	
30-Day Mortality	387 (45.3)	122 (50.8)	509 (46.5)	0.301
30-Day to 1-Year Mortality	194 (22.7)	47 (19.6)	241 (22.0)	
More than 1-Year Mortality	273 (32.0)	71 (29.6)	344 (31.4)	
Mortality—IHD COD, Excluding AMI (Total Events)	(*n* = 408)	(*n* = 122)	(*n* = 530)	
30-day mortality	51 (12.5)	15 (12.3)	66 (12.5)	0.624
30-Day to 1-Year Mortality	142 (34.8)	37 (30.3)	179 (33.8)	
More than 1-Year Mortality	215 (52.7)	70 (57.4)	285 (53.8)	

R *: rate per 100 person–years; Sig *: statistical significance; UA: unstable angina; NSTEMI: non-ST-segment elevation AMI; STEMI: ST-segment elevation AMI; AMI: acute myocardial infarction; IHD: ischemic heart disease; COD: cause of death; IHT: inter-hospital transfer; PCI +/− Stent: percutaneous coronary intervention, with or without stent insertion.

**Table 3:** Rates of primary diagnosis, principal procedure, comorbidities and mortality per 100 person–years for first-time admissions for acute coronary syndrome for Perth metropolitan admissions based on transfer status between 1 January 2007 and 31 December 2015.

Variables	Direct/No IHT	IHT	Total	Sig *
	*n* (R *)	*n* (R *)	*n* (R *)	*p* < 0.05
Primary Diagnosis (Total Events)	(*n* = 9570)	(*n* = 6787)	(*n* = 16357)	
UA	3071 (32.1)	1532 (22.6)	4603 (28.1)	<0.001
NSTEMI	4333 (45.3)	3645 (53.7)	7978 (48.8)	
STEMI	2166 (22.6)	1610 (23.7)	3776 (23.1)	
Percutaneous Coronary Interventions (PCI) (Total Events)	(*n* = 9570)	(*n* = 6787)	(*n* = 16357)	
Other Coronary Procedures	28 (0.3)	20 (0.3)	48 (0.3)	<0.001
PCI +/− Stent	5479 (57.3)	4916 (72.4)	10395 (63.6)	
Coronary Artery Bypass Grafting	347 (3.6)	473 (7.0)	820 (5.0)	
Comorbidities during the Same Admission (Total Events)	(*n* = 9570)	(*n* = 6787)	(*n* = 16357)	
Diabetes	1880 (19.6)	1163 (17.1)	3043 (18.6)	<0.001
Cardiac Arrest	163 (1.7)	75 (1.1)	238 (1.5)	0.002
Heart Failure	1180 (12.3)	652 (9.6)	1832 (11.2)	<0.001
Chronic Pulmonary Disease	79 (0.8)	33 (0.5)	112 (0.7)	0.010
Renal Insufficiency < 29 mL/min	275 (2.9)	137 (2.0)	412 (2.5)	0.001
Obese	211 (2.2)	133 (2.0)	344 (2.1)	0.282
In-Hospital Mortality (Total Events)	(*n* = 9570)	(*n* = 6787)	(*n* = 16357)	
Yes, Died in Hospital	418 (4.4)	91 (1.3)	509 (3.1)	<0.001
Post-Discharge Mortality by COD (Total Events)	(*n* = 2718)	(*n* = 1392)	(*n* = 4110)	
Non-IHD COD	1843 (67.8)	1005 (72.2)	2848 (69.3)	<0.001
AMI COD	608 (22.4)	246 (17.7)	854 (20.8)	
IHD COD, Excluding AMI	267 (9.8)	141 (10.1)	408 (9.9)	
Post-Discharge Mortality by Time Points (Total Events)	(*n* = 2718)	(*n* = 1392)	(*n* = 4110)	
30-Day Mortality	545 (20.1)	219 (15.7)	764 (18.6)	<0.001
30-Day to 1-Year Mortality	665 (24.5)	321 (23.1)	986 (24.0)	
More than 1-Year Mortality	1508 (55.5)	852 (61.2)	2360 (57.4)	

R*: rate per 100 person–years; Sig *: statistical significance; IHT: inter-hospital transfer; UA: unstable angina; NSTEMI: non-ST-segment elevation AMI; STEMI: ST-segment elevation AMI; AMI: acute myocardial infarction; IHD: ischemic heart disease; COD: cause of death; PCI +/− Stent: percutaneous coronary intervention, with or without stent insertion.

## Data Availability

The datasets generated and/or analysed during the current study are not publicly available due to strict requirements set out by the Human Ethics Research Committees regarding the storage and use of the data by authorised investigators. Due to the small cell size of the study we can only produce results in aggregate form to preserve anonymity and confidentially.

## Appendix A

Diagnosis codes for acute coronary syndromes from the International Classification of Diseases, Australian Modification 10th (ICD-10-AM) Edition and Procedural Codes for Primary Percutaneous Coronary Interventions from the Australian Classification of Health Interventions 10th Edition (ACHI-10) [24,28].

**International Classification of Diseases, Australian Modification 10th Edition**	
Acute MI ^1^	I21, I22
Unstable Angina	I20.0
**Australian Classification of Health Interventions, 10th Edition**	
CABG ^2^	38497-00, 38497-01, 38497-02, 38497-03, 38497-04, 38497-05, 38497-06, 38497-07, 38500-00, 38503-00, 38500-01, 38503-01, 38500-02, 38503-03, 38500-04, 38503-04, 38500-05, 38503-05, 90201-00, 90201-01, 90201-02, 90201-03
PCI ^3^ without stenting	38300-00, 38303-00, 38300-01, 38303-01
PCI ^3^ with stenting	38306-00, 38306-01, 38306-02, 38306-03, 38306-04, 38306-05
Cardiac Catheterisation	38200-00, 38203-00, 38206-00
Cardiac Angiography	38215-00, 38218-00, 38218-01, 38218-02
Other Coronary Procedures	38241-00, 38309-00, 38312-00, 38312-01, 38315-00, 38318-00 38318-01, 90218-00, 90218-01, 90218-02, 90218-03, 38505-00 38637-00, 38456-19, 38653-08
^1^ myocardial infarction; ^2^ coronary artery bypass graft; ^3^ percutaneous coronary intervention.	

## Appendix B

Identification of Comorbid Conditions by International Classification of Diseases, 10th Edition, Australian Modification [24].

**Comorbid Conditions**	**ICD-10-AM Codes**
Any Infectious or Parasitic Disease	A00.X-B99.X Z86.1
Any Neoplasms	C00.X-D29.0; D29.2-D48. Z85.X, Z86.0, Z51.0, Z51.1, Z51.2
Any Endocrine, Metabolic or Immune Disease	E00.X-E90.X, Z86.3
Any Blood Disease	D50.X-D99.X, R23.3, Z86.2
Any Mental Disorder	F00.X-F99.X, R44.X, R15.X, R32.X, Z86.5
Any Disease of the Nervous System or Sense Organs	B91, B94.X, E14.3X, E14.4X, G00.X-G80.X, G83.X-G99.X, H00.X-H95.X, R56.X, T85.0, T85.1, Z86.12, Z86.6, Z94.7, Z96.1, Z97.0, Z97.4, Z46.1
Any Diseases of the Circulatory System	I39.8, G81.X, I00.X-I84.9, I86.X-I99.X, K55.1, K55.9, Q22.X-Q23.X, R60.X, R47.X, R02, R45.7, R57.X, T82.X, T85.0, T85.1, G97.8, G97.9, Z86.7, Z94.1, Z94.3, Z95.X, Z45.0.
Any Diseases of the Respiratory System	J00.X-J99.X, Z87.0, Z94.2, Z94.3.
Any Diseases of the Digestive System	K00.X-K55.0, K55.2-K55.8, K56.0-K93.X, R17.X, R13, Z87.1X, Z94.4.
Any Diseases of the Genitourinary System	D29.1, E11.2X, I12.0, I13.1, I13.2, I13.9, I15.0, I15.1, N00.X-N99.X, R33, T83.X, Z87.4, Z94.0, Z96.0, Z99.2, Z46.6, Z49.X.
All Pregnancy, Childbirth and the Puerperium	O00.X-O99.X, Z87.5, Z32.X, Z33.X, Z34.X, Z35.X
Any Diseases of Skin or Subcutaneous Tissue	L00.X-L99.X, R21, Z87.2.
Any Diseases of the Musculoskeletal System and Connective Tissue	M00.X-M99.X, T84.X, Z87.3, Z96.XX.
Congenital Anomalies	Q00.X-Q99.X, Z87.7,
Any Symptoms, Signs and Ill-Defined Conditions	E03.5, G43.X, G44.X, G47.X, G93.3, L57.9, L85.8, L85.9, O28.X, R00.X, R01.X, R03.X-R07.X, R09.X-R12.X, R14, R16.X, R18.X-R20.X, R22.X-R23.X, R25.X-R27.X, R29.X-R30.X, R34-R36, R39.X, R40.X-R43.X, R45.X, R48.X-R55.X, R59.X, R61.X-R63.X, R68.X, R70.X-R99.X, Z87.8.
Any Injury or Poisoning	S00.X-T98.X, Z91.6, Z91.5.
Any Factor Influencing Health Status and Contact with Health Services	Z00.X-Z01.X, Z03.X-Z13.X, Z20.X-Z29.X, Z39.X, Z40.X-Z76.X, Z80.X-Z99.X

## Appendix C

Rates of all first-time admissions for Acute Coronary Syndrome in Western Australian between 1 January 2007 and 31 December 2015.

			**2007**	**2008**	**2009**	**2010**	**2011**	**2012**	**2013**	**2014**	**2015**
		**Person–Years**	*** *n* (†Pop*^n^*) (^‡^Rate)**	*** *n* (†Pop*^n^*) (^‡^Rate)**	*** *n* (†Pop*^n^*) (^‡^Rate)**	*** *n* (†Pop*^n^*) (^‡^Rate)**	*** *n* (†Pop*^n^*) (^‡^Rate)**	*** *n* (†Pop*^n^*) (^‡^Rate)**	*** *n* (†Pop*^n^*) (^‡^Rate)**	*** *n* (†Pop*^n^*) (^‡^Rate)**	*** *n* (†Pop*^n^*) (^‡^Rate)**
**All First-Time Admissions for ACS**		10,000 PY	2231 (2,106,139) (10.59)	2171 (2,171,700) (10.00)	2394 (2,240,250) (10.69)	2525 (2,290,845) (11.02)	2634 (2,353,409) (11.19)	2596 (2,425,507) (10.70)	2422 (2,486,944) (9.74)	2343 (2,517,608) (9.31)	2332 (2,540,672) (9.18)
**Sex**	**Male**	10,000 PY	1424 (1,061,703) (13.41)	1425 (1,094,894) (13.01)	1533 (1,129,438) (13.57)	1611 (1,154,064) (13.96)	1689 (1,185,050) (14.25)	1704 (1,223,614) (13.93)	1605 (1,254,322) (12.80)	1526 (1,266,894) (12.05)	1486 (1,276,698) (11.64)
	**Female**	10,000 PY	807 (1,044,436) (7.73)	746 (1,076,806) (6.93)	861 (1,110,812) (7.75)	914 (1,136,781) (8.04)	945 (1,168,359) (8.09)	892 (1,201,893) (7.42)	817 (1,232,622) (6.63)	817 (1,250,714) (6.53)	846 (1,263,974) (6.69)
**Age Categories**	**18–39 years**	10,000 PY	56 (664,579.8) (0.84)	65 (692,108) (0.94)	63 (721,124.2) (0.87)	69 (737,877.8) (0.94)	61 (759,428.8) (0.80)	67 (788,406.8) (0.85)	60 (812,349) (0.74)	39 (818,460.2) (0.48)	57 (820,681.6) (0.69)
	**40–49 years**	10,000 PY	238 (312,936) (7.61)	233 (319,462) (7.29)	280 (326,291) (8.58)	268 (331,769) (8.08)	297 (339,664) (8.74)	279 (348,097) (8.02)	275 (352,279) (7.81)	223 (352,549) (6.33)	222 (352,321) (6.30)
	**50–59 years**	10,000 PY	463 (271,099) (17.08)	459 (276,577) (16.60)	454 (283,168) (16.03)	487 (290,113) (16.79)	550 (298,183) (18.45)	475 (304,940) (15.58)	459 (311,027) (14.76)	460 (314,838) (14.61)	450 (317,069) (14.19)
	**60–69 years**	10,000 PY	518 (179,683) (28.83)	482 (188,757) (25.54)	545 (197,668) (27.57)	613 (206,391) (29.70)	577 (215,720) (26.75)	624 (223,304) (27.94)	600 (231,497) (25.92)	591 (237,551) (24.88)	545 (244,020) (22.33)
	**70–79 years**	10,000 PY	494 (108,340) (45.60)	494 (110,836) (44.57)	508 (113,943) (44.58)	510 (116,996) (43.59)	567 (120,746) (46.96)	558 (125,149) (44.59)	514 (129,259) (39.77)	501 (134,777) (37.17)	506 (139,722) (36.21)
	**80+ years**	10,000 PY	462 (63,991) (72.20)	438 (66,373) (65.99)	544 (68,650) (79.24)	578 (71,126) (81.26)	582 (73,962) (78.69)	593 (75,999) (78.03)	514 (77,914) (65.97)	529 (79,686) (66.39)	552 (81,786) (67.49)
**Residential Region**	**North Metro**	10,000 PY	529 (521,904) (10.14)	527 (538,186) (9.79)	583 (555,044) (10.50)	602 (566,661) (10.62)	657 (581,869) (11.29)	674 (598,095) (11.27)	547 (611,518) (8.94)	528 (618,424) (8.54)	545 (624,063) (8.73)
	**East Metro**	10,000 PY	331 (646,837) (5.12)	272 (668,176) (4.07)	338 (690,254) (4.90)	411 (708,164) (5.80)	387 (728,104) (5.32)	400 (751,439) (5.32)	354 (771,803) (4.59)	328 (783,807) (4.18)	349 (793,760) (4.40)
	**South Metro**	10,000 PY	814 (459,726) (17.71)	830 (476,498) (17.42)	888 (494,044) (17.97)	913 (506,307) (18.03)	980 (523,594) (18.72)	918 (543,328) (16.90)	897 (560,534) (16.00)	904 (571,692) (15.81)	851 (581,114) (14.64)
	**South West**	10,000 PY	195 (147,093) (13.26)	175 (151,495) (11.55)	172 (156,220) (11.01)	175 (159,873) (10.95)	188 (163,450) (11.50)	200 (168,774) (11.85)	194 (173,435) (11.19)	195 (176,511) (11.05)	197 (178,703) (11.02)
	**Great Southern**	10,000 PY	70 (55,271) (12.66)	78 (55,748) (13.99)	68 (56,411) (12.05)	68 (56,711) (11.99)	88 (57,237) (15.37)	81 (58,444) (13.86)	85 (59,445) (14.30)	78 (60,060) (12.99)	92 (60,290) (15.26)
	**Wheatbelt**	10,000 PY	96 (73,347) (13.09)	94 (73,935) (12.71)	98 (74,899) (13.08)	91 (75,540) (12.05)	86 (76,177) (11.29)	88 (77,469) (11.36)	80 (78,477) (10.19)	73 (78,506) (9.30)	84 (78,134) (10.75)
	**Goldfields**	10,000 PY	63 (55,407) (11.37)	55 (56,576) (9.72)	69 (57,636) (11.97)	92 (58,470) (15.73)	77 (59,425) (12.96)	62 (60,481) (10.25)	73 (61,096) (11.95)	58 (59,878) (9.69)	50 (58,521) (8.54)
	**Midwest**	10,000 PY	52 (60,885) (8.54)	60 (62,159) (9.65)	85 (63,361) (13.42)	90 (63,980) (14.07)	77 (64,985) (11.85)	90 (66,068) (13.62)	98 (66,929) (14.64)	84 (66,715) (12.59)	83 (66,000) (12.58)
	**Pilbara**	10,000 PY	49 (51,165) (9.58)	41 (53,670) (7.64)	49 (56,578) (8.66)	44 (58,912) (7.47)	46 (61,777) (7.45)	33 (63,606) (5.19)	44 (64,978) (6.77)	39 (64,297) (6.07)	35 (63,021) (5.55)
	**Kimberley**	10,000 PY	32 (34,504) (9.27)	38 (35,257) (10.78)	44 (35,803) (12.29)	38 (36,227) (10.49)	46 (36,791) (12.50)	48 (37,803) (12.70)	49 (38,729) (12.65)	53 (37,718) (14.05)	46 (37,066) (12.41)
	*** Unplaced**		0 (-) (-)	1 (-) (-)	0 (-) (-)	1 (-) (-)	2 (-) (-)	1 (-) (-)	1 (-) (-)	3 (-) (-)	0 (-) (-)
**Primary Diagnosis**	**UA**	10,000 PY	824 (2,106,139) (3.91)	736 (2,171,700) (3.39)	766 (2,240,250) (3.42)	883 (2,290,845) (3.85)	779 (2,353,409) (3.31)	686 (2,425,507) (2.83)	576 (2,486,944) (2.32)	520 (2,517,608) (2.07)	468 (2,540,672) (1.84)
	**NSTEMI**	10,000 PY	782 (2,106,139) (3.71)	814 (2,171,700) (3.75)	961 (2,240,250) (4.29)	1032 (2,290,845) (4.50)	1282 (2,353,409) (5.45)	1326 (2,425,507) (5.47)	1297 (2,486,944) (5.22)	1243 (2,517,608) (4.94)	1346 (2,540,672) (5.30)
	**STEMI**	10,000 PY	625 (2,106,139) (2.97)	621 (2,171,700) (2.86)	667 (2,240,250) (2.98)	610 (2,290,845) (2.66)	573 (2,353,409) (2.43)	584 (2,425,507) (2.41)	549 (2,486,944) (2.21)	580 (2,517,608) (2.30)	518 (2,540,672) (2.04)
**In Hospital Mortality**	**UA**	10,000 PY	2 (824) (24.27)	1 (736) (13.59)	2 (766) (26.11)	2 (883) (22.65)	2 (779) (25.67)	0 (686) (0.00)	2 (576) (34.72)	2 (520) (38.46)	3 (468) (64.10)
	**NSTEMI**	10,000 PY	17 (782) (217.39)	20 (814) (245.70)	29 (961) (301.77)	27 (1032) (261.63)	32 (1282) (249.61)	36 (1326) (271.49)	27 (1297) (208.17)	24 (1243) (193.08)	20 (1346) (148.59)
	**STEMI**	10,000 PY	57 (625) (912.00)	51 (621) (821.26)	44 (667) (659.67)	47 (610) (770.49)	47 (573) (820.24)	33 (584) (565.07)	34 (549) (619.31)	33 (580) (568.97)	32 (518) (617.76)
**Mortality**	**30-day**	1000 PY	120 (2231) (53.79)	101 (2171) (46.52)	113 (2394) (47.20)	121 (2525) (47.92)	127 (2634) (48.22)	118 (2596) (45.45)	98 (2422) (40.46)	100 (2343) (42.68)	100 (2332) (42.88)
	**30-days to 1-year**	1000 PY	115 (2231) (51.55)	121 (2171) (55.73)	156 (2394) (65.16)	141 (2525) (55.84)	141 (2634) (53.53)	157 (2596) (60.48)	148 (2422) (61.11)	134 (2343) (57.19)	146 (2332) (62.61)
	**More than 1-year**	1000 PY	555 (2231) (248.77)	489 (2171) (225.24)	481 (2394) (200.92)	442 (2525) (175.05)	368 (2634) (139.71)	316 (2596) (121.73)	208 (2422) (85.88)	130 (2343) (55.48)	32 (2332) (13.72)
**Primary Treatment**	**PCI ± Stent**	100 PY	1244 (2231) (55.76)	1275 (2171) (58.73)	1463 (2394) (61.11)	1525 (2525) (60.40)	1632 (2634) (61.96)	1622 (2596) (62.48)	1628 (2422) (67.22)	1553 (2343) (66.28)	1588 (2332) (68.10)
	**CABG**	100 PY	132 (2231) (5.92)	118 (2171) (5.44)	104 (2394) (4.34)	137 (2525) (5.43)	124 (2634) (4.71)	129 (2596) (4.97)	116 (2422) (4.79)	130 (2343) (5.55)	121 (2332) (5.19)
	**Other Coronary**	100 PY	1 (2231) (0.04)	8 (2171) (0.37)	11 (2394) (0.46)	8 (2525) (0.32)	4 (2634) (0.15)	2 (2596) (0.08)	11 (2422) (0.45)	14 (2343) (0.60)	14 (2332) (0.60)
**Transfer Status**	**Direct Admission**	100 PY	1266 (2231) (56.75)	1167 (2171) (53.75)	1313 (2394) (54.85)	1359 (2525) (53.82)	1286 (2634) (48.82)	1186 (2596) (45.69)	1147 (2422) (47.36)	1228 (2343) (52.41)	1247 (2332) (53.47)
	**Interhospital Transfer**	100 PY	965 (2231) (43.25)	1004 (2171) (46.25)	1081 (2394) (45.15)	1166 (2525) (46.18)	1348 (2634) (51.18)	1410 (2596) (54.31)	1275 (2422) (52.64)	1115 (2343) (47.59)	1085 (2332) (46.53)
**Admission Status**	**Emergency Admission**	100 PY	1944 (2231) (87.14)	1901 (2171) (87.56)	2148 (2394) (89.72)	2239 (2525) (88.67)	2448 (2634) (92.94)	2402 (2596) (92.53)	2244 (2422) (92.65)	2130 (2343) (90.91)	2063 (2332) (88.46)
	**Elective Admission**	100 PY	287 (2231) (12.86)	270 (2171) (12.44)	246 (2394) (10.28)	286 (2525) (11.33)	186 (2634) (7.06)	194 (2596) (7.47)	178 (2422) (7.35)	213 (2343) (9.09)	269 (2332) (11.54)
**MACSS 1 Year**	**0 MACSS**	100 PY	33 (2231) (1.48)	29 (2171) (1.34)	45 (2394) (1.88)	47 (2525) (1.86)	61 (2634) (2.32)	30 (2596) (1.16)	26 (2422) (1.07)	20 (2343) (0.85)	19 (2332) (0.81)
	**1 MACSS**	100 PY	140 (2231) (6.28)	197 (2171) (9.07)	216 (2394) (9.02)	248 (2525) (9.82)	274 (2634) (10.40)	224 (2596) (8.63)	165 (2422) (6.81)	157 (2343) (6.70)	146 (2332) (6.26)
	**2 MACSS**	100 PY	269 (2231) (12.06)	327 (2171) (15.06)	320 (2394) (13.37)	347 (2525) (13.74)	389 (2634) (14.77)	352 (2596) (13.56)	280 (2422) (11.56)	263 (2343) (11.22)	255 (2332) (10.93)
	**3+ MACSS**	100 PY	1789 (2231) (80.19)	1618 (2171) (74.53)	1813 (2394) (75.73)	1883 (2525) (74.57)	1910 (2634) (72.51)	1990 (2596) (76.66)	1951 (2422) (80.55)	1903 (2343) (81.22)	1912 (2332) (81.99)
**MACSS 5 Year**	**0 MACSS**	100 PY	14 (2231) (0.63)	9 (2171) (0.41)	21 (2394) (0.88)	21 (2525) (0.83)	25 (2634) (0.95)	11 (2596) (0.42)	11 (2422) (0.45)	6 (2343) (0.26)	8 (2332) (0.34)
	**1 MACSS**	100 PY	70 (2231) (3.14)	86 (2171) (3.96)	115 (2394) (4.80)	108 (2525) (4.28)	128 (2634) (4.86)	100 (2596) (3.85)	87 (2422) (3.59)	72 (2343) (3.07)	66 (2332) (2.83)
	**2 MACSS**	100 PY	141 (2231) (6.32)	177 (2171) (8.15)	172 (2394) (7.18)	196 (2525) (7.76)	212 (2634) (8.05)	188 (2596) (7.24)	146 (2422) (6.03)	154 (2343) (6.57)	143 (2332) (6.13)
	**3+ MACSS**	100 PY	2006 (2231) (89.91)	1899 (2171) (87.47)	2086 (2394) (87.13)	2200 (2525) (87.13)	2269 (2634) (86.14)	2297 (2596) (88.48)	2178 (2422) (89.93)	2111 (2343) (90.10)	2115 (2332) (90.69)
* *n*: number of patients; †Pop*^n^*: population for that year and demographic factor; ^‡^Rate: rate per X person–years, as per the person–years column UA: unstable angina; NSTEMI: non-ST-segment elevation AMI; STEMI: ST-segment elevation AMI; AMI: acute myocardial infarction; PCI +/− Stent: percutaneous coronary intervention, with or without stent insertion.; CABG: coronary artery bypass graft; MACSS: multipurpose Australian comorbidity scoring system.											
